# Serological response to influenza vaccination among adults hospitalized with community‐acquired pneumonia

**DOI:** 10.1111/irv.12622

**Published:** 2018-12-17

**Authors:** Caroline Quinn Pratt, Yuwei Zhu, Carlos G. Grijalva, Richard G. Wunderink, D. Mark Courtney, Grant Waterer, Min Z Levine, Stacie Jefferson, Wesley H. Self, Derek J. Williams, Lynn Finelli, Anna M. Bramley, Kathryn M. Edwards, Seema Jain, Evan J. Anderson

**Affiliations:** ^1^ Rollins School of Public Health Emory University Atlanta Georgia USA; ^2^ Department of Biostatistics Vanderbilt University School of Medicine Nashville Tennessee USA; ^3^ Department of Health Policy Vanderbilt University School of Medicine Nashville Tennessee USA; ^4^ Department of Medicine Northwestern University Feinberg School of Medicine Chicago Illinois USA; ^5^ Department of Emergency Medicine Northwestern University Feinberg School of Medicine Chicago Illinois USA; ^6^ University of Western Australia Perth WA Australia; ^7^ Centers for Disease Control and Prevention Atlanta Georgia USA; ^8^ Department of Emergency Medicine Vanderbilt University School of Medicine Nashville Tennessee USA; ^9^ Division of Hospital Medicine Monroe Carell Jr. Children‘s Hospital Department of Pediatrics Vanderbilt University School of Medicine Nashville Tennessee USA; ^10^ Division of Infectious Diseases Monroe Carell Jr. Children‘s Hospital Department of Pediatrics Vanderbilt University School of Medicine Nashville Tennessee USA; ^11^ Departments of Pediatrics and Medicine Emory University School of Medicine Atlanta Georgia USA

## Abstract

Ninety‐five adults enrolled in the Etiology of Pneumonia in the Community study with negative admission influenza polymerase chain reaction (PCR) tests received influenza vaccination during hospitalization. Acute and convalescent influenza serology was performed. After vaccination, seropositive (≥1:40) hemagglutination antibody titers (HAI) were achieved in 55% to influenza A(H1N1)pdm09, 58% to influenza A(H3N2), 77% to influenza B (Victoria), and 74% to influenza B (Yamagata) viruses. Sixty‐six (69%) patients seroconverted (≥4‐fold HAI rise) to ≥1 strain. Failure to seroconvert was associated with diabetes, bacterial detection, baseline seropositive titers for influenza B (Yamagata), and influenza vaccination in the previous season.

## 1 INTRODUCTION

Hospitalization provides an opportunity to administer influenza vaccine to unimmunized patients. Inpatient influenza vaccination is a quality measure for patients admitted from October to March.^1^ Although it has been shown to be safe,^2^ the immunogenicity and effectiveness are less clear. Data suggest that immune responses may be blunted due to age, admitting condition, or other comorbidities.^3^, ^4^, ^5^, ^6^, ^7^ We identified hospitalized adults with community‐acquired pneumonia (CAP) from the Centers for Disease Control and Prevention (CDC) Etiology of Pneumonia in the Community (EPIC) study who were influenza polymerase chain reaction (PCR) negative at admission and received influenza vaccine during their hospitalization, and assessed their serologic responses and the impact of preexisting conditions on the responses.^8^

## 2 METHODS

### 2.1 Patient population and study design

The EPIC study prospectively studied the incidence and etiology of CAP in adults hospitalized at five hospitals in Chicago and Nashville between January 1, 2010 and June 30, 2012. The study has been described elsewhere.^8^ Institutional review boards at each site and the CDC approved the study, and written informed consent was obtained. Detailed data were collected by interview and medical record review. Severity data were also collected (eg, pneumonia severity index [PSI], intensive care unit [ICU] admission, hospitalization duration).^8^ Prior seasonal influenza vaccination history was based on self‐report but verified by review of vaccine registry information, medical records, and pharmacies, when available. The vaccine for the 2010‐2011 and 2011‐2012 seasons contained influenza A/California/7/2009 (H1N1)‐like virus, A/Perth/16/2009 (H3N2)‐like virus, and influenza B/Brisbane/60/2008‐like virus. Blood and respiratory specimens were collected on admission for pathogen detection and tested using multiple modalities.

Patients included in this analysis were hospitalized, had radiographically confirmed pneumonia, and a naso/oropharyngeal (NP/OP) swab negative for influenza by PCR. Acute (collected upon enrollment) and convalescent (obtained 3‐10 weeks after enrollment) sera were tested for hemagglutination inhibition (HAI). Patients were also required to have received influenza vaccination during the current hospitalization (≥2 weeks before collection of convalescent serum).

### 2.2 Laboratory studies

Hemagglutination inhibition assays using standardized methods were performed on paired sera for the following strains circulating during 2010‐2012: A/California/07/2009 (H1N1)pdm09, A/Perth/16/2009 (H3N2), B/Brisbane/60/2008 (Victoria lineage), and B/Florida/4/2006 (Yamagata lineage) at CDC. HAI assays were performed using standardized methodologies.^8^

### 2.3 Serologic responses to influenza vaccination

A patient was considered to have seroconversion if a ≥ 4‐fold rise in HAI antibody titers occurred between acute and convalescent serum specimens with a convalescent titer of ≥40 for influenza A(H1N1)pdm09, A(H3N2), influenza B Victoria lineage, or influenza B Yamagata lineage.^8^, ^9^ Seropositive titers were defined as HAI ≥ 1:40.^10^

### 2.4 Statistical methods

Descriptive statistics included number (%) and median (interquartile range, IQR) for categorical and continuous variables, respectively. Demographic and clinical characteristics were compared between seroconversion and non‐seroconversion groups using Pearson's chi‐square test for categorical variables and Wilcoxon rank‐sum test for continuous variables.

To identify variables associated with non‐seroconversion in a setting with a relatively large number of covariates but a limited number of observations, we first identified a subset of potential variables using a least absolute shrinkage and selection operator approach (lasso) regression strategy.^11^ We then applied a multivariable logistic regression approach to assess the association between the subset of factors identified by the lasso, and the probability of non‐seroconversion.^12^ The study covariates evaluated in the lasso regression were age, gender, diabetes mellitus, smoking status, asthma, liver disease, kidney disease, cardiac disease, HIV, immunosuppression,^13^ chronic obstructive pulmonary disease (COPD), stroke, bacterial detection, non‐influenza respiratory virus detection, influenza vaccine receipt in previous season, prior 23‐valent pneumococcal polysaccharide vaccine receipt, hospitalization due to severe pneumonia illness, pre‐immunization HAI titer ≥1:40 for any influenza strain included in the vaccine, year of enrollment in the EPIC study, and hospital. The adjusted odds ratios (aORs) and 95% confidence intervals (CI) were calculated. Data analyses were conducted with SAS Software version 9.4 and R version 3.4.1. with glmnet package.

## 3 RESULTS

### 3.1 Patient population

Among the 2320 adults with radiographic pneumonia enrolled in the EPIC study, 95 (3.8%) patients met the selection criteria for this analysis. Patients were excluded for positive admission influenza PCR (153), no receipt of influenza vaccination while hospitalized (1824), vaccination twice while hospitalized or vaccinated <2 weeks before convalescent serology was obtained (84), and the absence of either acute or convalescent serology (164). Overall, the 95 that were vaccinated prior to hospital discharge were similar to the 2320 adults that were enrolled, although vaccine was more commonly administered at Hospital B than at Hospital D.

### 3.2 Factors associated with non‐seroconversion

In comparison with patients who seroconverted, patients who did not seroconvert were more likely to have diabetes mellitus (*P *=* *0.033) and a baseline HAI ≥ 40 for influenza B (Yamagata, *P *=* *0.026) in bivariate analyses (Table 1). Median age, immunosuppression, PSI score, ICU admission, and duration of hospitalization did not differ between those who seroconverted and those who did not.

**Table 1:** Distribution of epidemiological and clinical factors among patients who did and did not have seroconversion, defined as at least one ≥4–fold rise in HAI antibody titers between acute and convalescent serum specimens

Variable	All patients (n = 95)	Seroconversion (n = 66)	Non‐Seroconversion (n = 29)	*P*‐value
	n (%)	n (%)	n (%)	
Gender				
Female	40 (42.1)	31 (47.0)	9 (31.0)	0.147
Race/Ethnicity				
Hispanic	15 (15.8)	11 (16.7)	4 (13.8)	0.214
White	40 (42.1)	30 (45.5)	10 (34.5)	
Black	36 (37.9)	24 (36.4)	12 (41.4)	
Asian	4 (4.2)	1 (1.5)	3 (10.3)	
Smoking	30 (31.6)	20 (30.3)	10 (34.5)	0.886
Study Hospital				
Hospital A	36 (37.9)	27 (40.9)	9 (31.0)	0.726
Hospital B	22 (23.2)	15 (22.7)	7 (24.1)	
Hospital C	16 (16.8)	11 (16.7)	5 (17.2)	
Hospital D	5 (5.3)	4 (6.1)	1 (3.5)	
Hospital E	16 (16.8)	9 (13.6)	7 (24.1)	
Year of study enrollment				
2010	32 (33.7)	21 (31.8)	11 (37.9)	0.363
2011	49 (51.6)	37 (56.1)	12 (41.4)	
2012	14 (14.7)	8 (12.1)	6 (20.7)	
Receipt of influenza vaccine in prior season	21 (22.1)	11 (16.7)	10 (34.5)	0.054
Receipt of both influenza and PPV 23 vaccines	57 (60.0)	39 (59.1)	18 (62.1)	0.850
Previous pneumonia admission	27 (28.4)	18 (27.3)	9 (31.0)	0.708
Asthma	29 (30.5)	19 (28.8)	10 (34.5)	0.579
Coronary artery disease or heart failure	28 (29.5)	20 (30.3)	8 (27.6)	0.789
Diabetes mellitus	20 (21.1)	10 (15.2)	10 (34.5)	0.033*
COPD	14 (14.7)	9 (13.6)	5 (17.2)	0.648
Chronic kidney disease	11 (11.6)	6 (9.1)	5 (17.2)	0.253
Chronic oral steroid use	9 (9.5)	6 (9.1)	3 (10.3)	0.807
Liver disease	7 (7.4)	7 (10.6)	0 (0.0)	0.068
Stroke	7 (7.4)	4 (6.1)	3 (10.3)	0.462
HIV+ (with CD4 > 200/14%)	2 (2.1)	1 (1.5)	1 (3.4)	0.546
Non‐skin cancer	9 (9.5)	6 (9.1)	3 (10.3)	0.848
Immunosuppression	8 (8.4)	4 (6.1)	4 (13.8)	0.211
Baseline HAI titer ≥ 40: A(H1N1)pdm09	23 (24.2)	16 (24.2)	7 (24.1)	0.991
Baseline HAI titer ≥ 40: A(H3N2)	24 (25.3)	14 (21.2)	10 (34.5)	0.170
Baseline HAI titer ≥ 40: B (Victoria)	37 38.9)	23 (34.8)	14 (48.3)	0.217
Baseline HAI titer ≥ 40: B (Yamagata)	56 (58.9)	34 (51.5)	22 (75.9)	0.026*
Identification of a bacterial pathogen	14 (14.7)	7 (10.6)	7 (24.1)	0.087
Identification of a viral pathogen	32 (33.7)	24 (36.4)	8 (27.6)	0.405
ICU Admission	20 (21.1)	14 (21.2)	6 (20.7)	0.954
*Continuous Variables*		Median (IQR)	Median (IQR)	
Age (years)	54.0 (44.0–71.0)	54.0 (42.0–65.0)	55.00 (49.0–71.0)	0.505
BMI	27.5 (23.7–32.4)	26.9 (23.7–32.0)	28.6 (24.2–33.4)	0.676
PSI score	70.0 (48.0–102.0)	68.0 (48.0–101.0)	71.0 (48.0–106.0)	0.597
Duration of hospitalization	3.0 (2.0–5.0)	3.5 (2.0–5.0)	3.0 (2.0–5.0)	0.674

COPD – chronic obstructive pulmonary disease; PPV23 – 23 valent pneumococcal polysaccharide vaccine; HAI – hemagglutination inhibition; ICU – intensive care unit; IQR – interquartile range; BMI – body mass index; PSI – pneumonia severity index.

* *P*‐value < 0.05

The lasso regression model identified factors including diabetes mellitus, prior receipt of seasonal influenza vaccine, immunosuppressive conditions, bacterial pathogen detection, liver disease, baseline seropositive titers to influenza B (Yamagata), and study enrollment during 2012. In the final multivariable logistic regression model, only diabetes mellitus, prior receipt of seasonal influenza vaccine, bacterial pathogen detection, and baseline seropositive titers to influenza B (Yamagata) were significantly associated with non‐seroconversion after in‐hospital influenza vaccination (Table 2).

**Table 2:** Adjusted odds ratios (OR) with 95% confidence intervals (CI) for non‐seroconversion to any influenza strain after influenza vaccination

Risk factor^a^	Adjusted OR (95% CI)
Bacterial detection	4.04 (1.01‐16.22)
Receipt of influenza vaccine in previous season	3.60 (1.16‐11.19)
Diabetes mellitus	3.59 (1.14‐11.3)
Seropositive titer against influenza B (Yamagata)	3.39 (1.17‐9.88)
Immunosuppression	2.18 (0.41‐11.52)
Enrolled in 2012	1.96 (0.49‐7.75)

a History of liver disease was not included in the final model due to collinearity.

### 3.3 Seroconversion to individual influenza strains

Sixty‐six (69%, 95% CI 59%‐79%) of these 95 patients seroconverted to ≥1 influenza virus strain: 44% to influenza A(H1N1)pdm09, 46% to influenza A(H3N2), 34% to influenza B (Victoria), and 22% to influenza B (Yamagata). Seropositive titers after vaccination were achieved in 55% (95% CI 45‐65) of patients to influenza A(H1N1)pdm09, 58% (95% CI 48‐68) to influenza A(H3N2), 77% (95% CI 68, 86) to influenza B (Victoria), and 74% (95% CI 665, 83) to influenza B (Yamagata) viruses (Table 3). Among those with a baseline HAI titer ≥ 40 for individual strains, after vaccination 35% (8/23) of these individuals developed a ≥ 4 fold HAI rise to influenza A(H1N1)pdm09, 29% (7/24) to influenza A(H3N2), 8% (3/37) to influenza B (Victoria), and 14% (8/56) to influenza B (Yamagata).

**Table 3:** Acute and convalescent serology titers ≥40 by strain

Strain	Acute serology ≥ 40, n (%)	95% CI	Convalescent serology ≥ 40, n (%)	95% CI
Influenza A (H1N1)pdm09	23 (24)	16, 32	52 (55)	45, 65
Influenza A (H3N2)	24 (25)	17, 34	55 (58)	48, 68
Influenza B (Victoria)	37 (39)	29, 49	73 (77)	68, 86
Influenza B (Yamagata)	56 (59)	49, 69	70 (74)	65, 83

## 4 DISCUSSION

Influenza vaccination during CAP hospitalization resulted in seroconversion to ≥1 influenza virus strains in 69% of the 95 patients. We lacked a directly comparable control group of non‐hospitalized adults, but overall seroconversion rates observed here were suboptimal.^4^, ^5^ Potential reasons for suboptimal seroconversion include a reduced immune response with acute infection or the use of immunosuppressive medications (eg, corticosteroids). Since we have no comparative data to patients hospitalized with an acute noninfectious illness, distinguishing between effects of acute infection versus other possibilities is difficult.

We had anticipated that age, mild immunosuppression, or measures of CAP severity (eg, PSI score, duration of hospitalization, and ICU admission) might explain differences in seroconversion, but these associations were not observed (Table 1). Prior receipt of influenza vaccine and diabetes has been previously identified as risk factors for non‐seroconversion after influenza vaccination in people who were not acutely ill.^3^, ^4^

Seropositive titers were achieved for >50% of influenza A strains and about 75% of influenza B strains following vaccination. Importantly, this was shortly after inclusion of the new influenza A(H1N1)pdm09 strain in the vaccine. More potent vaccines could improve immunogenicity among adults at risk of poor serological responses. High‐dose influenza vaccine is licensed by the US Food and Drug Administration (FDA) and is recommended for prevention of influenza A and B in persons ≥65 years of age. High‐dose influenza vaccine has demonstrated improved rates of seroconversion and vaccine efficacy in those ≥65 years of age compared with the traditional inactivated standard dose influenza vaccines, which were most commonly used during this study.^14^, ^15^ Additionally, the FDA has licensed MF59 as an adjuvant for influenza vaccination in the elderly. Evaluation of the safety and immunogenicity of these new vaccines could benefit hospitalized patients with CAP (many of whom are <65 years of age) at risk of non‐seroconversion.

This study has several limitations, including design as a *post‐hoc* analysis of data from a prospective observational study. Vaccine manufacturers and vaccination policies may have differed across the centers. Differences were observed between several centers in the percentage of hospitalized patients that received influenza vaccine, although seroconversion did not differ by hospital. The study was restricted to patients with inpatient vaccination and available acute and convalescent serology. Differences between patients who returned for convalescent serology and those that did not return could have impacted the results. Patients who were PCR negative at admission may have been subsequently infected with influenza during the window before collection of convalescent sera, resulting in HAI seroconversion due to natural infection and not vaccination. Influenza vaccination status was determined by self‐report with verification when possible. Estimates in this study lack some statistical power to detect differences between the groups due to the small sample size. Finally, due to the small numbers, the immune responses were evaluated in aggregate rather than examining responses to each antigen separately.

Despite a lower seroconversion rate after influenza vaccination for adults who received the vaccine during an acute hospitalization for CAP than might be observed among healthy adult outpatients, hospitalization represents an important opportunity to vaccinate patients who may not return for vaccination after discharge; and almost 70% of our study patients did seroconvert to ≥1 strain.^1^, ^2^ Seropositive titers were achieved in >50% of patients against both influenza A strains and >70% against both influenza B strains. Identifying adults at risk of not seroconverting with standard influenza vaccination could focus future prospective clinical trials of new influenza vaccination strategies (eg, adjuvanted or high‐dose influenza vaccine). Improving both vaccination rates and vaccine immunogenicity is important in preventing the substantial burden of influenza morbidity and mortality in adults.

## CONFLICT OF INTEREST

WHS reports research funding from Merck & Co Inc, Pfizer, Gilead Sciences, and Ferring Pharmaceuticals, and consultant fees from Cempra Pharmaceuticals, Ferring Pharmaceuticals, and BioTest AG. EJA reports prior consulting for AbbVie, research funding from MedImmune, Regeneron, Pfizer, Merck &Co Inc, PaxVax, and Sanofi Pasteur.
